# Public Perceptions, Beliefs and Experiences of Fostering and Adoption: A National Qualitative Study in South Africa

**DOI:** 10.1111/chso.12122

**Published:** 2015-04-17

**Authors:** Tamsen J Rochat, Zitha Mokomane, Joanie Mitchell, The Directorate

**Affiliations:** ^1^ Africa Centre for Health and Population StudiesUniversity of KwaZulu‐NatalKwaZulu‐NatalSouth Africa; ^2^ Department of PsychologyStellenbosch UniversityStellenboschSouth Africa; ^3^ Section of Child and Adolescent PsychiatryDepartment of PsychiatryUniversity of OxfordOxfordUK; ^4^ Human Sciences Research CouncilPretoriaSouth Africa; ^5^ Department of SociologyUniversity of PretoriaPretoriaSouth Africa; ^6^ Adoptions and International Social ServicesNational Department of Social DevelopmentPretoriaSouth Africa

**Keywords:** adoption, fostering, perceptions, qualitative, South Africa

## Abstract

In South Africa, rates of adoption remain low while the number of fostered children continually rises. Little is known about the public perceptions, beliefs and experiences that inform decisions to either foster or adopt in South Africa. This qualitative research explored these issues among a national sample of childless adults, biological parents, kin and non‐kin fostering parents and prospective and successful adopters. Fostering is driven predominantly by access to subsidies but is also informed by socio‐cultural beliefs. Low adoption rates are influenced by an absence of subsidies, poor access to quality adoptive services and a lack of information about adoption.

## Introduction

The systemic effects of HIV and poverty result in many South African children losing parental care and requiring social assistance (Monasch and Boerma, 2004; Pequegnat and others, 2012). Both orphaning and abandonment are on the increase (Meintjes and others, 2009), particulary in rural provinces where services are sparse (de Villiers and Giese, 2008). Encouragingly, fostering has increased over the last decade (Mokomane and Rochat, 2011), but unfortunately the numbers of children (in particular HIV‐infected children) placed in residential care has also increased (Moses and Meintjes, 2010).

Data on children in residential care are sparse and inconsistent; available estimates suggest that at least 15 590 children are currently placed in registered residential care facilities, likely an underestimation given the proliferation of unregistered homes (Meintjes and others, 2007). Residential care placements are commonly as a result of either: abuse and neglect (30%), abandonment (24%) or orphaning (11%) (The Presidency, 2009). The majority of HIV‐affected children are absorbed within extended or re‐constituted families (Hosegood, 2009); these family systems are increasingly strained (Biemba and others, 2010). For HIV‐infected or affected and abandoned or abused children whose families are unwilling or unsuitable to care for them, adoption likely has considerable benefits over residential care.

Fostering and foster care are different in African contexts, compared to Europe and the United States (Grant and Yeatman, 2012; Monasch and Boerma, 2004). In general in those settings, foster care is not considered permanent (though it may be long‐term in specific legal cases). The State retains guardianship of the child during this interim period of care. Foster care is typically used until a child can be reunited with a parent, is permanently adopted or reaches adulthood. In African settings, fostering, and in particular kin fostering is considered a more permanent placement (Blackie, 2014). Children move between and within families to increase access to resources and care, using less formal fostering processes and with less concern for legal process or protection (Abebe, 2010).

South Africa's Children's Act (No 38 of 2005) promotes family and kinship care whenever possible and residential care as a last resort. In promotion of family fostering, the Social Assistance Act (Act No 13, 2004) provides fostering parents with a monthly subsidy of ZAR860 or USD75. Significant implementation challenges exist (see Matthias and Zaal, 2009). Fostering placements are formalised through legal custodial processes, needing review every 2 years by court process (Hall and Proudlock, 2011) and significant backlogs have been documented. Adoption requires extensive initial screening and court procedures, but is distinctive from fostering in its requirement for the termination of parental rights. Unlike most international settings, there are no adoption subsidies or tax credits for adopting parents, and no incentive systems for social welfare or adoption agencies (DeVooght and others, 2011). It is plausible that the absence of adoption subsidies together with cultural beliefs which discourage termination of parental rights, results in low adoption rates (Blackie, 2014). Research in the early 2000s found a willingness among families to provide adoptive placements for HIV‐affected and vulnerable children (Townsend and Dawes, 2007); however, this has not translated into increased adoptions.

The aim of this research was to explore the perceptions and beliefs that drive decisions to foster or adopt among South Africans in both urban and rural settings.

## Methodology

This research was part of a larger study commissioned by the Directorate of Adoptions and International Social Services at the South African Department of Social Development (Mokomane and Rochat, 2011). Ethical approval was received from the Research Ethics Committee of the Human Science Research Council of South Africa and the Biomedical Ethics Review Board of the University of KwaZulu‐Natal.

Data were collected (2010–2011) in four of the nine provinces in South Africa. Recruitment used snowball purposive sampling from initial contact lists sourced from: social welfare databases; established child placement support services and support groups; community leadership forums and non‐profit organisations; which all offer services for fostered and adopted children.

We constituted six homogenous focus group (FG) types including:

1. Kin Foster parents: Blood‐related family members fostering a child;
2. Non‐Kin Foster parents: Non‐blood‐related family members fostering a child;
3. Adoptive parents: Adoption procedures completed in the last 5 years;
4. Prospective adoptive parents: Prospective adopters on waiting lists;
5. Non‐adoptive parents: Parents of biological children only;
6. Childless adults: Childbearing adults, in and outside relationships who do not have children.

We repeated these six types of FG in four geographical locations (Gauteng, Eastern Cape; KwaZulu‐Natal and Limpopo) completing 17 FGs with 102 participants. While FGs for all childcare groups were successfully constituted in Gauteng (South Africa's economic hub with the highest population density), in other regions, sufficient numbers to constitute the *adoptive parents* and *prospective adoptive parents* groups proved a challenge, likely reflective of low levels of adoption across the country. Similarly, it was difficult to locate non‐kin foster parents in areas in the Eastern Cape and KwaZulu‐Natal, likely because of its rural population and cultural norms supporting kin care. In these cases, in‐depth interviews were completed. FGs and interviews used a standardised FG guide. All data were tape‐recorded and transcribed, and translated into English. The FG topics explored the following three areas:

1. Personal preferences, perceptions and beliefs about fostering and adoption,
2. Suggested interventions to increase adoption,
3. Public awareness and knowledge of the purpose/processes of fostering and/or adoption.

A thematic analysis was used with themes surfacing from the data as little to no research existed within this topic area in South Africa. Through repeated readings of the transcripts by two independent researchers, initial thematic codes were identified. These themes were organised into categories, and were consolidated into an initial coding scheme and organised using NVivo software (QSR International, Melbourne, Australia). FG and interviews were then analysed using between‐group analysis (Onwuegbuzie and others, 2009) to determine the salience of categories within and across groups. This was determined by using FG content, assessing the length and depth of discussion around the theme, and the number of times an issue was raised both within and across groups.

The results are presented with illustrative quotations. To ensure anonymity names are removed, childcare type, role, gender and age range are identified.

## Results

Three important contextual thematic categories emerged and framed perceptions and decisions about adoption and fostering in the data:

1. The social context: socioeconomic and socio‐cultural influences
2. The service context: access to and quality of adoptive services
3. The knowledge context: public awareness and access to information

Between‐group analysis showed that the salience of themes A and B differed between two main clusters of participants in a fairly pragmatic way:

Cluster 1: groups which included fostering, non‐adoptive, biological and childless parent participants gave greater salience to theme A;

Cluster 2: groups which included prospective and adoptive parent participants gave greater salience to theme B although some aspects of theme A were raised.

Theme C was salient for all groups although less so compared to themes A and B. Cluster 1 tended to have slightly lower socioeconomic status than cluster 2, who in turn were more educated and Caucasian.

### The social context: socioeconomic and socio‐cultural influences

Two themes dominated: socioeconomic access to financial resources to care for children; and to a lesser extent socio‐cultural issues were important in determining preferences for adoption or fostering. This theme was most salient for cluster 1.

#### Socioeconomic influences

The financial needs of fostering families and their inability to care for children without a subsidy, as would be the case if they adopted, emerged as the main reason for opting for fostering over adoption in South Africa.

Finance is a major problem, no matter what you say about adoption, if people don't have the money they just can't do it (Commissioner of Child Welfare, Male, KwaZulu‐Natal, 50+ years).

There was consensus across groups that introduction of subsidies would likely increase adoption.

The government [if it aims to increase adoption] must give subsidies to those who are willing and waiting to adopt, but are unable to do so for financial constraints, it just makes sense (Biological parent, female, Gauteng, 40–50 years).

Emotional investment in the child resulted in a desire to provide for the child's needs, which in turn led to monetary concerns and needs. The love and nurturing that fostering parents provide could not be substituted with money, and was not the direct result of money. While subsidies were seen as a means to support basic needs, thus making it feasible to care for the child, they were not perceived as payment for childcare itself, which was considered more valuable.

I can easily afford the love a child needs, it costs nothing and it is worth everything to the child. I can promise to be there and make sure that I am, much more than a volunteer in a dormitory with 20 kids. I can't afford the schooling, the food and all the things they need. I am willing to work but I can't find work, that's why I need a grant, not because I want money for myself, I need to take care of this child, I love this child so how can you expect me to not want to give this child everything (Non‐kin foster parent, female, KwaZulu‐Natal, 30–40 years).

Discussions frequently included spirited remarks on the inappropriateness of suspicions about fostering parent's intentions regarding financial assistance.

I know there are people who misuse it, I am not stupid, as a community leader I have had to deal with that a few times, but its disappointing when they say people are just misusing, there are many more honest people in my community than those who are doing wrong things, it is those same honest people who come and report those doing wrong. It is neighbours and teachers. The very same people who receive grants who report the ones who misuse grants, that's the majority (Community Leader, male, KwaZulu‐Natal, 50+ years).

Historical references to apartheid were common to stress the context of poverty as the driver of parental need for assistance, not greed.

Maybe they will trust that we are wanting to take care of our own children and that we are not lazy and looking for money for nothing. We have been doing this for a long time you know, taking care of our children. All through the struggle we did what we had to, we moved our children around, even as a child I lived with very little but I was loved and felt I belonged. We shared it out and we did the best we could. Now I must do the same for my community and for the price we paid for freedom we should not have to do it alone anymore (Community Advisory Board member, female, KwaZulu‐Natal, 40–50 years).

Beyond basic needs, educational and health care were cited as the main concerns requiring financial assistance. When asked whether provision of services either in lieu of or in combination with financial subsidies, a model frequently applied in international settings (Pinkerton and Muhangi, 2009), would be acceptable, the majority of participants considered this acceptable, but only if services were of a high quality and available within reasonable waiting periods.

Education, especially good education is so expensive but it really can change a life. There are always costs, so if you assure me this child can get a good education for free, that would make it much more affordable for me to adopt (Community leader, male, Gauteng, 30–40 years).

#### Socio‐cultural influences

This highlighted socio‐cultural beliefs which influence how families understand responsibilities towards kin and non‐kin children. Three important issues were raised that influence decisions either to foster or adopt.

Firstly, extended kin families are considered the most appropriate source of care, support and security in the absence of parental care, regardless of any governmental policy.

Adoption, hmmm, I don't know, I don't really see the point – in our culture these children are our responsibility and we know that – a piece of paper would not change that so what is the point? (Kin foster parent, female, KwaZulu‐Natal, 30–40 years).

As this kin care is culturally normative, it does not need to be mandated by legal processes.

No, the child is already mine, I've never thought of changing it, or taking legal steps (Kin foster parent, female, Limpopo, 50+ years).

Secondly, non‐kin care placements which involve the severing of parental rights, or reject the father's or the family of origin's right to claim the child, could have damaging social consequences for children.

In a case of a kin, you should not make that child hate his father's family. If you take that child and adopt him you can only cause problems between families (Biological parent, female, Gauteng, 20–30 years).

Thirdly, specific beliefs were raised by both urban and rural participants regarding ancestral world views, specifically that children deprived of their roots would lose contact with their ancestors, with unpleasant, punitive consequences for the future happiness of the child.

When you are born, there are certain things that ancestors require of us. They know who our child is and where he is. Just imagine if you adopt a Biyela child and join the child to the Mthembu's. There will be war between the Biyela and Mthembu ancestors, both ancestors will fight over who owns the child (Biological parent, male, Gauteng, 40–50 years).

Ritualistic practices to appease ancestors and allow non‐kin placement of children requires family of origin information, which raises concerns in regards to achieving this with abandoned children, when no information is available.

There is an ancestral family that one must consider before adopting a child. How will I conduct the necessary ceremonies without knowing this child's ancestral roots? If I don't do this, then I will bring problems to this child and to my own family (Biological parent, female, Gauteng, 30–40 years).

### The service context: access and quality of adoptive services

Among participants in Cluster 2, constituted mostly by parents who had already decided to or successfully adopted, theme B was most salient and related to the lack of access to good quality, efficient and friendly adoptive services. Two key areas of concern were raised: firstly lengthy waiting periods; and the performance, or lack thereof, of the assigned social worker.

Long waiting periods led to descriptions of the adoptive system as unkind, unfair and un‐empathetic to the needs of adoptive parents.

It is a very long process…it feels unfair, and hurtfully so, after you have made a commitment to take a child (Adoptive parent, female, Gauteng, 40–50 years).

The stress of the wait and the wondering… yes, they need to do this and go through it carefully, that's fine. But the waiting is the hardest part, I'm not sure they understand how hard that part is (Prospective adoptive parent, female, Gauteng, 30–40 years).

Participants found social worker lack of empathy, competency and availability problematic. Inconsistencies in interpretation and implementation of adoptive processes among social workers in different settings, and over time were common experiences. The majority believed that, if left to the social worker alone, their adoption would not have happened.

The most difficult thing in our adoption was getting the social worker to do something (Adoptive parent, female, Kwazulu‐Natal, 40–50 years).

I've thought about adoption before but I don't think about it anymore because of what I have heard—the experience and the long application process. It would be too much for me to handle (Childless adult, female, Limpopo, 20–30 years).

They told me that before I could take these children in, I had to go to court – so for their and my sake ‐ I ended up fostering them as the adoption process was just too long (Non‐kin foster parent, female, Gauteng, 50+ years).

Participants expressed outrage that timelines for placement were determined by the social workers availability and not the child's need for placement, in particular when adopting from residential care. For many participants, the only viable solution had been to procure a privately paid social worker.

We ended up employing a private social worker to do the job because the government social worker was incapable, many social workers don't always know what to do. I think they are often not clear on the legal process [and] social workers on the ground don't know exactly the process to follow. But private social workers are motivated and know exactly the legal process to follow (Adoptive parent, female, KwaZulu‐Natal, 40–50 years).

Some prospective adopters had developed reservations following delays.

Obviously there are not that many children in need of homes then, so I might as well explore other options, I thought I was doing the right thing looking to adoption, but if there are no kids available, well then I might as well look at surrogacy or something (Childless adult, female, KwaZulu‐Natal, 30–40 years).

### The knowledge context: public awareness and access to information

Participants reported access to information was sparse, poorly distributed and often relied on chance. Misperceptions about the process of adoption and stereotypes about eligibility were common.

I had a friend whose sister was working at a children's home and she told me about fostering and adoption (Successful adoptive parent, female, Gauteng, 30–40 years).

We were talking at the office when this lady mentioned that she knew a woman who looked after babies until they got adopted. She gave me her number (Successful adoptive parent, female, Eastern Cape, 30–40 years).

Many participants self‐selected out of adoption based on misperceptions about their eligibility, including financial and marital status requirements and a poor understanding of the process by which adoption decisions and placements would be made. Common misperceptions included beliefs that one had to be legally married, own a property or be infertile to qualify.

Yes, I have that you have to be employed, financially stable, married and have a house to meet the requirements of adoption but for us in the rural areas those requirements are scarce (Biological parent, female, Limpopo, 20–30 years).

I've heard if you are unable to have children you can apply and take someone else's child and make it your own (Biological parent, female, Limpopo, 20–30 years).

## Discussion

Socioeconomic factors, including not having access to resources to care for children, socio‐cultural beliefs and common misperceptions about financial requirements for eligibility to adopt were key barriers to adoption in this sample. The merits of provision of social security grant/subsidies aimed at improving the welfare of children in South Africa are debated in the literature (Adato and Bassett, 2009; Richter, 2009). There is little consensus regarding approach (cash/strengthened services) or whether conditionality will prove helpful or harmful in the context of significant poverty (Giese, 2009; Lund, 2011). In this research fostering and adoptive parents call for consideration to be given, to the monetary value of the childcare provided by either fostering or adoptive parents, which likely far exceeds the monetary value of grants. It is well documented in the literature (Desmond and Gow, 2001; Swales, 2006) that family care models bear lower monetary costs, and generally result in better child outcomes, when compared with residential care (see Bos and others, 2011 for a recent review of literature).

Our finding that service provision may be an acceptable substitute for financial subsidies, as is common in other international settings, is interesting, but not without problems. The acceptability of services in lieu of subsidies was conditional on these being salient (health and education) accessible and of high quality. Current preferences for cash subsidies may thus, at least in part, be related to low confidence in the quality and availability of such services through public health educational systems. The provision of services and subsidies for adopting parents is also intertwined with the provision of the current subsidy to fostering parents. It is likely that many fostering parents, in the absence of the foster care grant, may be disqualified as potential adopters as a result of their lack of employment and/or regular income. While the ‘cash versus care’ debates continue (Hall and Proudlock, 2011), we illustrate here growing frustration among families at a grass‐root level regarding access to social assistance and services.

While less important than socioeconomic determinants, socio‐cultural influences play an important role in decisions to foster or adopt, a finding which is not uncommon in other parts of Africa, and in qualitative research in South Africa (Blackie, 2014). More generally, the termination of parental rights to facilitate adoption (national or otherwise) is a common challenge in the adoption literature, with cultural world views having been shown to influence fostering decisions and outcomes in other settings (Brown and others, 2011; Cushing and Greenblatt, 2009; Taylor, 2010). Within this cultural milieu, a uniform approach of advocating for legal adoptive processes over formal or informal fostering may prove fruitless and unpopular (Riley, 2012). Culturally, longer term kin fostering may be as appropriate and beneficial as adoption in international contexts, with kin‐fostered children experiencing the benefits of permanent placement without being adopted (Cuddeback, 2004). Kin care has been shown to have benefits in terms of stability of placement in African countries, despite the low level of resources and support offered to them (Farmer, 2009; Miller and others, 2006; Schofield and others, 2007; Wegar, 2000).

The proliferation of fostering, and socioeconomic and socio‐cultural preferences for it raises child protection, fiscal and administrative challenges (United Nations, 2009). While fostering may be culturally sensitive, its informality can increase the vulnerability of children (particularly those placed with distant relatives or in poverty‐stricken household) to exploitation, abuse and neglect (Heymann and others, 2012; Malinga and Ntshwarang, 2011; Tshoose, 2010). Alternatively when fostering is formalised to reduce these risks, it becomes impractical and unsustainable in poorly resourced settings from both a legislative and administrative perspective. Research has shown that increases in lapsed foster care grants in 2010 are attributed predominantly to lapsing court orders as a result of the significant burden the ongoing review of the foster care grants places on the judicial system (Hall and Proudlock, 2011).

While increased adoptions may reduce this burden, an increase is unlikely in the absence of adoption subsidies. Potential savings in human services costs and reduced judicial burden should be considered in cost estimates for the provision of adoption subsidies (Hansen, 2008). While socioeconomic factors were the strongest driver in decisions to foster or adopt in this research, whether providing adoption subsidies or strengthened services would negate the socio‐cultural issues raised, or simply bring them to the fore, remains unknown.

Cultural beliefs encouraging clan ownership of children may benefit the majority of children, but disadvantage some, in particular abandoned children in residential care. Some research in South Africa (Blackie, 2014) found that traditional belief systems make provisions for rituals and practices to address concerns around ancestry and parental rights issues. While inter‐country adoption is controversial, it must be considered for these children as they have a low chance of being adopted nationally (Mokomane and others, 2011; Rotabi and Gibbons, 2012) in particular if their family of origin is unknown thus adding a cultural disadvantage to their chances of domestic adoption, making international adoption more beneficial than long‐term residential care (Bartholet, 2010; Davies, 2011).

The central role of the social worker may account for their being frequently blamed for delays in administration and court proceedings. Common perceptions of lack of empathy and the absence of case management strategies to promote timely placement for children are concerning and served as a disincentive for considering adoption. Steep attrition rates following initial adoption enquiries are common internationally and the quality of the first enquiry has been shown to mediate later frustrations when administrative delays emerge (Wilson and others, 2005). Improving recruitment and information services could improve relationships with service providers and mediate the negative effects of time delays. Likewise simplifications in administration which facilitate placements and reduce waiting periods, while still providing adequate protection for children, are warranted (Ainsworth and Hansen, 2011; Selwyn and others, 2006).

Concerns over inadequate investments in human resources are well documented (Matthias and Zaal, 2009). Dawes (2011) reports that human resources shortages cause significant delays in processing, screening and placement given there are only 13 773 registered social workers in South Africa. Their time is generally consumed with administration processes as opposed to service provision in their specialist area. Service delivery is likely equally affected by poor implementation strategies, a lack of minimum standards and inadequate resource investments (Matthias and Zaal, 2009). In international settings, the use of incentive systems and the privatisation of placement services have been implemented to increase the quality and speed of placements, with varying success (Fink and others, 2011; Selwyn and Sempik, 2011).

Agency level incentives, the use of private sector human resources and accountability for minimum standard with regard to time delays have shown some success in developed countries and warrant consideration (DeVooght and others, 2011; Fink and others, 2011). Improved service orientation among those who serve as gatekeepers and first contacts for prospective adopters has also been shown to positively influence adoption rates, as has specialised evidence‐based training of all those involved in the screening and placement process, including social workers and court officials (Cushing and Greenblatt, 2009; Lee and others, 2010; van de Luitgaarden, 2009; Wegar, 2000).

Among the general public, awareness and knowledge about adoption was low and access to information poor. South Africa had until recently invested few resources in promoting and or disseminating information on adoption. Increasing public's knowledge of adoption has been shown to improve attitudes towards adoption in other settings in Africa (Ezugwu and others, 2002; Omosun and Kofoworola, 2011). Important recent steps to address this issue include the formation of the National Adoption Coalition of South Africa, which draws together public and private sector resources to increase awareness about adoption in South Africa (www.adoptioncoalitionsa.org). It includes the establishment of a website and toll‐free call‐centre service to provide centralised, accurate information to the public.

## Conclusions

Socioeconomic concerns and the quality of adoptive services, along with socio‐cultural beliefs and a lack of public knowledge about adoption, are the most important determinates of decisions to adopt or foster. Engagement with cultural leaders may assist in the development of specific campaigns to address cultural barriers (such as social‐cultural or ancestral beliefs) to adoption, in particular for the most vulnerable children, being those without family of origin information or options. Increasing awareness towards adoption, in particular among families who have means to adopt without access to a subsidy, is a key priority.

## References

[chso12122-bib-0001] Abebe T . 2010 Beyond the ‘orphan burden’: understanding care for and by AIDS‐affected children in Africa. Geography Compass 4: 460–474. doi:10.1111/j.1749‐8198.2009.00301.x.

[chso12122-bib-0002] Adato M , Bassett L . 2009 Social protection to support vulnerable children and families: the potential of cash transfers to protect education, health and nutrition. AIDS Care 21: 60–75. doi:10.1080/09540120903112351. 22380980 10.1080/09540120903112351 PMC2903773

[chso12122-bib-0003] Ainsworth F , Hansen P . 2011 The experience of parents of children in care: the human rights issue. Child & Youth Services 32: 9–18. doi:10.1080/0145935x.2011.553578.

[chso12122-bib-0004] Bartholet E . 2010 International adoption: the human rights position. Global Policy 1: 91–100. doi:10.1111/j.1758‐5899.2009.00001.x.

[chso12122-bib-0005] Biemba G , Beard J , Brooks B , Bresnahan M , Flynn D . 2010 The scale, scope and impact of alternative care for OVC in developing countries: a critical review paper. Centre for Global Health and Development, Boston University Available at http://www.bu.edu/cghd/files/2009/12/The-Scale-Scope-and-Impact-of-Alternative-Care-for-OVC-Full-Report-2.12.10.pdf [Accessed 2 March 2015].

[chso12122-bib-0006] Blackie D . 2014 Child abandonment and adoption in the context of African ancestral beliefs in contemporary urban South Africa: Fact Sheet on Child Abandonment Research in South Africa. National Adoption Coalition South Africa (NACSA) Available at http://www.adoptioncoalitionsa.org/wp-content/uploads/2014/05/Fact-Sheet-Research-on-Child-Abandonment-in-South-Africa_Final2.pdf [Accessed 2 March 2015].

[chso12122-bib-0007] Bos K , Zeanah CH , Fox NA , Drury SS , McLaughlin KA , Nelson CA . 2011 Psychiatric outcomes in young children with a history of institutionalization. Harvard Review of Psychiatry 19: 15–24. doi:10.3109/10673229.2011.549773. 21250893 10.3109/10673229.2011.549773 PMC3445019

[chso12122-bib-0008] Brown JD , George N , Arnault DS , Sintzel J . 2011 Cultural worldviews of foster parents. Journal of Family Social Work 14: 21–42. doi:10.1080/10522158.2011.523869.

[chso12122-bib-0009] Cuddeback GS . 2004 Kinship family foster care: a methodological and substantive synthesis of research. Children and Youth Services Review 26: 623–639. doi:10.1016/j.childyouth.2004.01.014.

[chso12122-bib-0010] Cushing G , Greenblatt SB . 2009 Vulnerability to foster care drift after the termination of parental rights. Research on Social Work Practice 19: 694–704. doi:10.1177/1049731509331879.

[chso12122-bib-0011] Davies M . 2011 Intercountry adoption, children's rights and the politics of rescue. Adoption & Fostering 35: 50–62. doi:10.1177/030857591103500406.

[chso12122-bib-0012] Dawes A . 2011 The Children's HIV and AIDS Scorecard 2011: Monitoring South Africa's Response to Children and HIV and AIDS. Childrens Rights Centre: Durban Available at http://www.childrensrights.org.za/index.php/documents/network-materials?download=122:scorecard2011 [Accessed 2 March 2015].

[chso12122-bib-0013] Desmond C , Gow J . 2001 The Cost Effectiveness of Six Models of Care for Orphans and Vulnerable Children in South Africa. UNICEF & University of Natal: Durban, South Africa Available at http://www.unicef.org/evaldatabase/files/SAF_01-801.pdf [Accessed 2 March 2015].

[chso12122-bib-0014] DeVooght K , Malm K , Vandivere S , McCoy‐Roth M . 2011 Trends in Adoptions from Foster Care in the Wake of Child Welfare Reforms (Vol. Analysis No. 4). Fostering Connections: Washington, DC Available at http://s3.amazonaws.com/zanran_storage/www.fosteringconnections.org/ContentPages/2466875399.pdf [Accessed 2 March 2015].

[chso12122-bib-0015] Ezugwu FO , Obi SN , Onah HE . 2002 The knowledge, attitude and practice of child adoption among infertile Nigerian women. Journal of Obstetrics & Gynaecology 22: 211–216. doi:10.1080/01443610120113463. 12521712 10.1080/01443610120113463

[chso12122-bib-0016] Farmer E . 2009 Placement stability in kinship care. Vulnerable Children and Youth Studies 4: 154–160. doi:10.1080/17450120902887871.

[chso12122-bib-0017] Fink J , de Jong A , Langan M . 2011 New challenges or different opportunities? Voluntary adoption agencies and the shifting terrain of childcare services Voluntary Sector Review 2: 177–191. doi:10.1332/204080511x583841.

[chso12122-bib-0018] Giese S . 2009 Spotlighting the relationship between social welfare services and cash transfers within social protection for children. Vulnerable Children and Youth Studies 4: 1–5. doi:10.1080/17450120903012974.

[chso12122-bib-0019] Grant MJ , Yeatman S . 2012 The relationship between orphanhood and child fostering in sub‐Saharan Africa, 1990s–2000s. Population Studies 66: 279–295. doi:10.1080/00324728.2012.681682. 22607126 10.1080/00324728.2012.681682 PMC3473157

[chso12122-bib-0020] Hall K , Proudlock P . 2011 Orphaning and the Foster Child Grant: A Return to the “Care or Cash” Debate. Children Count Brief. Children's Insitutute, University of Cape Town: Cape Town Available at http://www.childrencount.ci.org.za/uploads/Policy%20brief%20FCG_final.pdf [Accessed 2 March 2015].

[chso12122-bib-0021] Hansen ME . 2008 The value of adoption. Adoption Quarterly 10: 65–87. doi:10.1300/J145v10n02_03.

[chso12122-bib-0022] Heymann J , Sherr L , Kidman R . 2012 Protecting Childhood in the AIDS Pandemic: Finding Solutions that Work. Oxford University Press: Oxford.

[chso12122-bib-0023] Hosegood V . 2009 The demographic impact of HIV and AIDS across the family and household life‐cycle: implications for efforts to strengthen families in sub‐Saharan Africa. AIDS Care 21: 13–21. doi:10.1080/09540120902923063. 22380974 10.1080/09540120902923063 PMC2758218

[chso12122-bib-0024] Lee BR , Shaw TV , Gove B , Hwang J . 2010 Transitioning from group care to family care: child welfare worker assessments. Children and Youth Services Review 32: 1770–1777. doi:10.1016/j.childyouth.2010.07.021.

[chso12122-bib-0025] van de Luitgaarden GMJ . 2009 Evidence‐based practice in social work: lessons from judgment and decision‐making theory. British Journal of Social Work 39: 243–260. doi:10.1093/bjsw/bcm117.

[chso12122-bib-0026] Lund F . 2011 A step in the wrong direction: linking the South Africa Child Support Grant to school attendance. Journal of Poverty and Social Justice 19: 5–14. doi:10.1332/175982711x559118.

[chso12122-bib-0027] Malinga T , Ntshwarang PN . 2011 Alternative care for children in Botswana: a reality or idealism? Social Work and Society 9: 1–13.

[chso12122-bib-0028] Matthias CR , Zaal FN . 2009 Supporting familial and community care for children: legislative reform and implementation challenges in South Africa. International Journal of Social Welfare 18: 291–298. doi:10.1111/j.1468‐2397.2008.00586.x.

[chso12122-bib-0029] Meintjes H , Moses S , Berry L , Mampane R . 2007 Home Truths: The Phenomenon of Residential Care for Children in a Time of AIDS. Childrens Institute, University of Cape Town & Centre for the Study of AIDS, University of Pretoria: Cape Town Available at https://open.uct.ac.za/bitstream/handle/11427/4094/CI_researchreports_residentialcare_2007-06.pdf?sequence=1 [Accessed 2 March 2015].

[chso12122-bib-0030] Meintjes H , Hall K , Marera DH , Boulle A . 2009 Orphans of the AIDS epidemic? The extent, nature and circumstances of child‐headed households in South Africa. AIDS Care 22: 40–49. doi:10.1080/09540120903033029. 20390479 10.1080/09540120903033029 PMC2840873

[chso12122-bib-0031] Miller CM , Guskin S , Subramanian SV , Rajaraman D , Heymann SJ . 2006 Orphan care in Botswana's working households: growing responsibilities in the absence of adequate support. American Journal of Public Health 96: 149–1435. doi:10.2105/ajph.2005.072280. 10.2105/AJPH.2005.072280 PMC1522103 16809585

[chso12122-bib-0032] Mokomane Z , Rochat TJ , Directorate Adoptions and International Social Services . 2011 Adoption in South Africa: trends and patterns in social work practice. Child & Family Social Work 17: 347–358. doi:10.1111/j.1365‐2206.2011.00789.x.

[chso12122-bib-0033] Monasch R , Boerma JT . 2004 Orphanhood and childcare patterns in sub‐Saharan Africa: an analysis of national surveys from 40 countries. AIDS 18: 55–65. doi:10.1097/00002030‐200406002‐00007. 10.1097/00002030-200406002-00007 15319744

[chso12122-bib-0034] Moses S , Meintjes H . 2010 Positive care? HIV and residential care for children in South Africa. African Journal of AIDS Research 9: 107–115. doi:10.2989/16085906.2010.517475. 25860520 10.2989/16085906.2010.517475

[chso12122-bib-0035] Omosun AO , Kofoworola O . 2011 Knowledge, attitude and practice towards child adoption amongst women attending infertility clinics in Lagos State, Nigeria. African Journal of Primary Health Care & Family Medicine 3: 1–8. doi:10.4102/phcfm.v3i1.259.

[chso12122-bib-0036] Onwuegbuzie AJ , Dickinson WB , Leech NL , Zoran AG . 2009 A qualitative framework for collecting and analyzing data in focus group research. International Journal of Qualitative Methods 8: 1–21.

[chso12122-bib-0037] Pequegnat W , Bell CC , Allison S . 2012 The role of families among orphans and vulnerable children in confronting HIV/AIDS in Sub‐Saharan Africa In Family and HIV/AIDS. Pequegnat W Bell CC (eds.). Springer: New York, NY; 173–194.

[chso12122-bib-0038] Pinkerton J , Muhangi D . 2009 Linking social welfare development with cash transfers and education to promote child wellbeing – what we know and what we need to know. Vulnerable Children and Youth Studies 4: 55–67. doi:10.1080/17450120903131188.

[chso12122-bib-0039] Richter LM . 2009 Can and should cash transfers be linked to social welfare? Vulnerable Children and Youth Studies 4: 72–76. doi:10.1080/17450120903128598.

[chso12122-bib-0040] Riley N . 2012 Making Families Through Adoption. Sage: London.

[chso12122-bib-0041] Rotabi K , Gibbons J . 2012 Does the Hague convention on intercountry adoption adequately protect orphaned and vulnerable children and their families? Journal of Child and Family Studies 2: 106–119. doi:10.1007/s10826‐011‐9508‐6.

[chso12122-bib-0042] Schofield G , Thoburn J , Howell D , Dickens J . 2007 The search for stability and permanence: modelling the pathways of long‐stay looked after children. British Journal of Social Work 37: 619–642. doi:10.1093/bjsw/bch275.

[chso12122-bib-0043] Selwyn J , Sempik J . 2011 Recruiting adoptive families: the costs of family finding and the failure of the inter‐agency fee. British Journal of Social Work 41: 415–431. doi:10.1093/bjsw/bcq075.

[chso12122-bib-0044] Selwyn J , Frazer L , Quinton D . 2006 Paved with good intentions: the pathway to adoption and the costs of delay. British Journal of Social Work 36: 561–576. doi:10.1093/bjsw/bch272.

[chso12122-bib-0045] Swales DM . 2006 Applying the Standards: Improving Quality Childcare Provision in East and Central Africa. Save the Children: London Available at http://www.savethechildren.org.uk/sites/default/files/docs/ApplyingTheStandards_1.pdf [Accessed 2 March 2015].

[chso12122-bib-0046] Taylor L . 2010 Resurrecting parents of legal orphans: un‐terminating parental rights. Virginia Journal of Social Policy & the Law 17: 318–372.

[chso12122-bib-0047] The Presidency . 2009 Situational Analysis of Children in South Africa. UNICEFL: Pretoria Available at http://www.thepresidency.gov.za/docs/pcsa/gdch/situation-analysis.pdf [Accessed 2 March 2015].

[chso12122-bib-0048] Townsend L , Dawes A . 2007 Intentions to care for children orphaned by HIV/AIDS: a test of the theory of planned behavior. Journal of Applied Social Psychology 37: 822–843. doi:10.1111/j.1559‐1816.2007.00188.x.

[chso12122-bib-0049] Tshoose C . 2010 The impact of HIV/AIDS regarding informal social security: issues and perspectives from a South African context. PER: Potchefstroom Electronic Law Journal 13: 408–447. doi:10.4314/pelj.v13i3.63676.

[chso12122-bib-0050] United Nations . 2009 Guidelines for the alternative care of children (Vol. GE.09‐14213 (E) 160609): United Nations Available at http://www.unicef.org/protection/alternative_care_Guidelines-English.pdf [Accessed 2 March 2015].

[chso12122-bib-0051] de Villiers N , Giese S . 2008 A Review of Childrens Access to Employment Based Contributory Social Insurance Benefits. UNICEF & The Department of Social Development: Pretoria Available at http://www.unicef.org/southafrica/SAF_officialstatement_aidakpglpp.pdf [Accessed 2 March 2015].

[chso12122-bib-0052] Wegar K . 2000 Adoption, family ideology, and social stigma: bias in community attitudes, adoption research and practice. Family Relations 49: 363–370. doi:10.1111/j.1741‐3729.2000.00363.x.

[chso12122-bib-0053] Wilson JB , Katz J , Geen R . 2005 Listening to Parents: Overcoming Barriers to the Adoption of Children from Foster Care. Malcom Weiner Centre for Social Policy, Donaldson Adoption Institure & The Urnam Institute Available at http://www.hks.harvard.edu/ocpa/pdf/Listening%20to%20Parents.pdf [Accessed 2 March 2015].

